# Geographical Accessibility of Pediatric Inpatient, Nephrology, and Urology Services in Europe

**DOI:** 10.3389/fped.2020.00395

**Published:** 2020-07-21

**Authors:** Nicolas Terliesner, Dariusz Lesniowski, Alexandra Krasnikova, Martin Korte, Mirjam Terliesner, Marcus A. Mall, Katalin Dittrich

**Affiliations:** ^1^Department of Pediatric Pulmonology, Immunology and Intensive Care Medicine, Charité Universitätsmedizin Berlin, Berlin, Germany; ^2^Department of Pediatric Nephrology, University Hospital Leipzig, Leipzig, Germany; ^3^Department of Oral and Maxillofacial Surgery, Charité Universitätsmedizin Berlin, Berlin, Germany; ^4^Medical Faculty, Ruhr-Universität Bochum, Bochum, Germany; ^5^Medical Faculty, Universität Leipzig, Leipzig, Germany; ^6^Berlin Institute of Health, Berlin, Germany; ^7^Deutsche Stiftung Organtransplantation, Frankfurt, Germany

**Keywords:** access, geographical accessibility, travel time, travel distance, nephrology, hemodialysis

## Abstract

**Background:** Although many children with diseases of the kidneys and the urinary tract may not tolerate long journeys, the number of facilities that provide specialized care for these patients is limited. Therefore, the geographical accessibility of the required health services is critical especially in this patient group. We have analyzed the geographical accessibility of pediatric inpatient and nephro-urology services in Germany, Ireland, and the United Kingdom (UK).

**Methods:** This study introduces a model to compare countries or regions regarding the geographical accessibility of their health services. We calculated the geodesic distances, travel distances, and travel time by car from evenly distributed random points to the nearest facilities that provide pediatric inpatient or nephro-urology outpatient services (pediatric inpatient ward, urology clinic, nephrology clinic, hemodialysis unit). The results were weighted by population density. We compared the three countries with regard to the accessibility of the named services.

**Results:** Weighted median travel times from the random points to the nearest pediatric inpatient ward are < 30 min in all countries. Weighted travel times to the nearest point of pediatric service are shortest in the UK (median <50 min) and longest in Ireland (median <90 min), regardless of the type of service (*p* < 0.0001). Non-weighted travel times to the nearest pediatric inpatient ward and hemodialysis unit, however, are shorter in Germany than in the UK (*p* < 0.0001).

**Conclusions:** There is a surprising disparity between the travel times to the nearest facility with pediatric nephro-urology service in these three industrialized European countries. Reasons may be differences in the geographical distribution of the population, the focus of the health care system, and a different degree of clinical networking.

## Introduction

The geographical accessibility of health care facilities is a main factor of both the availability of services and their affordability (1). There have been studies that have shown worse outcomes for adult patients who live further away from health care facilities than for those who live closer (2). Some data suggests that this may also apply to pediatric patients, in developing as well as in developed countries (3). For children with diabetes, a shorter travel time to the point of pediatric diabetes service is associated with a lower HbA1c (4). Successful transition of patients with sickle cell disease from pediatric to adult care is more likely for patients who live close to the adult sickle cell care center than for those living further away (5). Furthermore, even in an urban area longer travel distances to a pediatric consultant outpatient clinic are associated with a lower rate of attendance (6). Besides the impact on treatment and outcome of pediatric patients, longer travel distances to health care facilities naturally involve higher costs (e.g., direct travel costs, missed work hours, child care costs). Accordingly, families with lower socioeconomic status (SES) seem to be less able to afford long distance travels to a specialized pediatric clinic than those with higher SES (7).

Children with diseases involving the kidneys or the urinary tract comprise a marginal patient group that requires highly specialized medical care. The limited number of facilities within a health care system providing such specialized care also limits their geographical accessibility. The specific characteristics of this patient group, however, suggest that these patients do not tolerate long journeys to the outpatient clinic. Examples are newborns or young infants with congenital anomalies of the kidneys and the urinary tract (CAKUT), or patients visiting a hemodialysis unit several times a week, who cannot tolerate further travel-related absence from school or further restrictions in their social life. There is little data published on the impact of the geographical accessibility of specialized nephro-urology services on the outcome of pediatric patients. Children living further away from transplant centers are less likely to receive a kidney transplant compared to those living closer (8, 9). More data, however, is available on adults. Facilitating access to hemodialysis for adults with chronic kidney disease by setting up local hemodialysis units in non-urban areas leads to a higher rate of patients who receive renal replacement therapy (10, 11). Adult patients with kidney transplantation who live far away from the transplant center have an increased risk of post-transplantation death compared to those who live closer (12).

Especially in industrialized countries with sufficient resources, health care systems thus are expected to provide specialist treatment in pediatric nephrology and urology within short distance from home. There have been studies on the geographical accessibility of pediatric inpatient services in the regions of Japan (13, 14). To our knowledge, the geographical accessibility of specialized pediatric nephro-urology services has not been investigated yet. Therefore, the aims of this study were to determine and compare the geographical accessibility of such services in industrialized countries, and to discover possible reasons for differences in the accessibility of services between these countries. We used geographical information system (GIS)-based analyses to calculate distances and travel times from patients' homes to institutions that provide pediatric nephro-urology services (general pediatric inpatient ward, pediatric urology outpatient clinic, nephrology outpatient clinic, and hemodialysis unit for pediatric patients) in three industrialized countries in Europe. The results were then related to country- and health care system-related characteristics. We chose to compare Germany, Ireland, and the UK (listed in alphabetical order), as these countries offer a comparable standard of living, but differ concerning the geographical distribution of their population and the way their health care system is organized. Furthermore, on these countries sufficient data was available.

## Materials and Methods

One aim of this study was to keep all calculations reproducible and transferable. Therefore, we utilized only publicly accessible data and applications.

### Data Acquisition

Data about hospital services was mainly collected from public webpages. This included hospital websites, websites of physicians' associations and collaborations, press releases, job descriptions, and data published by health care providers and related institutes for statistics (waiting time statistics, referral statistics, and bed occupancy rates). Where the data about hospital services from public sources was not sufficient, the hospitals were contacted by phone or e-mail. Acquisition of data on local services in Germany was undertaken from 09/2018 to 11/2018, in Ireland from 12/2018 to 01/2019, and in the UK from 03/2019 to 05/2019. Supplementary Tables 1, 2 show the hospital services that we acquired data on and the criteria that we applied to evaluate whether a hospital provided the named service. An aim was to include all types of health service that frequently treat pediatric patients with diseases of the kidneys and the urinary tract in a non-intensive care setting. We included general pediatric inpatient wards in our analyses because a large part of children with kidney diseases (e.g., patients with CAKUT or steroid-sensitive nephrotic syndrome) may be treated there. As this study focuses on access to specialized pediatric nephrology services, the term “urology outpatient clinic” is defined as a clinic that treats patients with CAKUT. This is why, additionally to designated pediatric urology outpatient clinics, all facilities providing a pediatric nephrology outpatient clinic were also considered to provide a pediatric urology outpatient service. Coordinates of the geographical points were assigned using data from the website https://www.koordinaten-umrechner.de/ (accessed 09/2018 to 05/2019). Information about the Nomenclature of Territorial Units for Statistics (NUTS) 2016 regions (geography, social and macroeconomic indicators) was obtained from the Eurostat online database. The data sets generated and analyzed during the current study are available from the corresponding author on reasonable request.

### Data Analysis

Data was analyzed using Python v3.7. The European Terrestrial Reference System 1989 Lambert Azimuthal Equal Area Europe (EPSG:3035) was employed as the coordinate reference system for all calculations, as it is recommended for purposes where true area representation is required (15). In order to avoid the distance bias caused by islands, these were excluded from all calculations. We generated one random point per km^2^ geographical area for all three countries (Germany: 351,868 points; Ireland: 70,473 points; UK: 245,104 points). There is a slight difference between the exact number of random points and the related country area (in km^2^) due to the method of first generating random points, then keeping only the points within the countries' boundaries. The distribution of the points was checked visually by scatter plotting them. We identified the NUTS (classification of 2016) level 3 polygon that each random point belongs to, thereby assigning the corresponding population density to each random point. We then calculated the geodesic (shortest path between two points on the surface of the earth) distance from every random point to every point of pediatric service (general pediatric inpatient ward, urology outpatient clinic, nephrology outpatient clinic, hemodialysis unit) according to Karney, 2013 (16). We assigned the nearest provider of each of these types of service and the geodesic distance toward it to every random point. Then, using openrouteservice, we calculated travel time and travel distance by car from each random point to the nearest provider of each service. Openrouteservice, a service developed by The Heidelberg Institute for Geoinformation Technology that is based on OpenStreetMap, determines travel times and distances independently of daytime and live traffic data. All available routing services that include traffic information (e.g., Google Maps Directions API, Bing Maps Distance Matrix API) are subject to charges when travel distance and time matrices for a sample of points with a relevant size are requested. That is why we avoided these services in favor of reproducibility. Instead, we used traffic indices by INRIX, Inc. and TomTom International BV, which are publicly accessible, to compare cities regarding traffic load. These traffic indices are available only for areas in which a prolongation of travel due to traffic congestion is noticeable and thus were relevant only for analyses that involved a high amount of service providers in congested urban areas (i.e., distance, travel distance and travel time to pediatric hemodialysis units). Therefore, all cities with a pediatric hemodialysis unit were included in the comparison of traffic indices (for 82% of which a traffic index was available). Due to the limit in travel data requests per time when using openrouteservice, we calculated travel time and distance from a random sample of about 28% of the random points of each country (Germany: 100,000 points; Ireland: 20,000 points; UK: 70,000 points) to the nearest provider for each type of pediatric service. Since a possibly relevant fraction of the total number of hospitals in Ireland (30%) is privately funded, we performed all analyses on Ireland twice, once including private hospitals and once excluding them. This was not necessary for Germany and the UK. For every type of service in each country, mean, median, standard deviation, weighted mean, weighted median, and weighted standard deviation of geodesic distance, travel distance by car, and travel time by car from the random points to the points of service were calculated. The population density of the NUTS 3 region that each random point belongs to was employed as the weighting factor. To test for normal distribution, Shapiro-Wilk test was applied. As none of the data sets were normally distributed, we used Kruskal-Wallis test (to compare all three countries' data sets for each type of service) and Mann-Whitney *U* test (for pairwise comparison of the countries' data sets for each type of service) to test for statistical significance of the observed results.

## Results

The geographical distribution of the random points generated for each country as well as the sample of these random points that was used for the travel distance and travel time analyses shows no clusters or gaps (Supplementary Figures 1, 2).

### Weighted Geodesic Distance, Travel Distance, and Time to Nearest Point of Service

**Figure 1:**
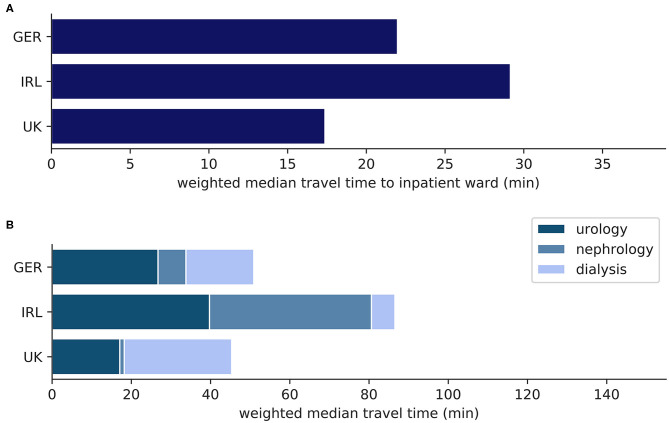
**(A)** Weighted median travel time (min) from random points to nearest pediatric inpatient ward; **(B)** Weighted median travel time (min) from random points to nearest facility with pediatric nephro-urology service; inpatient: pediatric inpatient ward; urology: pediatric urology outpatient clinic; nephrology: pediatric nephrology outpatient clinic; dialysis: pediatric hemodialysis unit.

**Figure 2:**
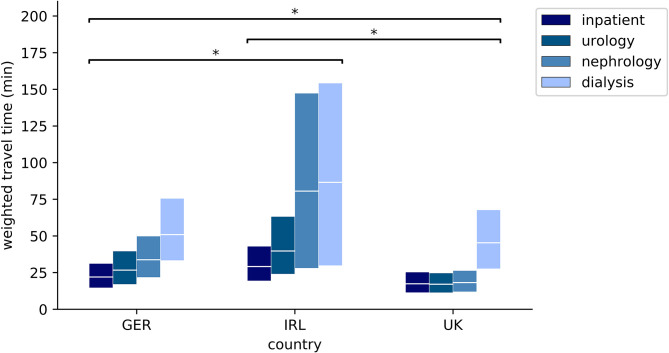
Weighted quartiles of travel time (min) from random points to nearest facility with pediatric service (upper box edge: 75th percentile, middle line: median; lower box edge: 25th percentile); *p < 0.0001; inpatient: pediatric inpatient ward; urology: pediatric urology outpatient clinic; nephrology: pediatric nephrology outpatient clinic; dialysis: pediatric hemodialysis unit.

Median and mean geodesic distance, travel distance by car, and travel time by car from the random points to the nearest pediatric inpatient ward and to the nearest points of pediatric nephro-urology service (pediatric urology outpatient clinic, pediatric nephrology outpatient clinic, pediatric hemodialysis unit) in each country, weighted by the population density of the NUTS 3 region that each random point belongs to, are shown in Tables 1, 2 and plotted in Figures 1, 2. In all three countries, the weighted median travel time to the nearest pediatric inpatient ward is <30 min. Concerning the nephro-urology services, in all three countries the weighted travel times and distances are the longest to the nearest hemodialysis unit, second longest to the nearest nephrology clinic, and shortest to the nearest urology clinic. With the exception of urology clinics in the UK, weighted travel time, and distance to the nearest pediatric nephro-urology service are longer than to the nearest pediatric inpatient ward. There is a considerable difference in weighted geodesic distances, travel distances, and travel times from the random points to the nearest pediatric services between the three countries. Weighted distances and times are shortest in the UK (median: 17–45 min), longer in Germany (median: 27–51 min), and longest in Ireland (median: 37–87 min), no matter whether private hospitals in Ireland are included. This applies to all types of service, and each of these observed differences between the countries is highly significant (*p* < 0.0001 according to both Kruskal-Wallis test and Mann-Whitney *U*-test).

**Table 1:** Weighted median and mean (parentheses) geodesic distance (km), travel distance by car (km), and travel time by car (min) from random points to nearest pediatric inpatient ward; Ireland (boxes): privately funded hospitals excluded (upper), included (lower); *p < 0.0001.

	**Distance (km)**			**Travel distance (km)**			**Travel time (min)**		
**Country**	**GER**	**IRL**	**UK**	**GER**	**IRL**	**UK**	**GER**	**IRL**	**UK**
Inpatient*	12.85 (15.23)	26.70 (28.89)	11.81 (15.16)	15.62 (18.58)	27.40 (31.51)	13.76 (17.24)	21.98 (24.02)	29.15 (33.07)	17.37 (19.96)
		26.49 (28.45)			27.17 (30.99)			28.33 (32.34)	

**Table 2:** Weighted median and mean (parentheses) geodesic distance (km), travel distance (km), and travel time (min) by car from random points to nearest facility with pediatric nephro-urology service (urology: urology outpatient clinic; nephrology: nephrology outpatient clinic; dialysis: hemodialysis unit); Ireland (boxes): privately funded hospitals excluded (upper), included (lower); *p < 0.0001.

	**Distance (km)**			**Travel distance (km)**			**Travel time (min)**		
**Country**	**GER**	**IRL**	**UK**	**GER**	**IRL**	**UK**	**GER**	**IRL**	**UK**
Urology*	17.78 (21.38)	40.04 (47.07)	11.65 (15.75)	21.42 (26.16)	40.79 (50.41)	13.29 (17.35)	26.72 (30.04)	39.72 (47.08)	17.08 (19.95)
		37.35 (40.80)			37.92 (46.02)			37.26 (43.77)	
Nephrology*	25.84 (30.98)	125.51 (156.39)	12.66 (16.92)	30.96 (37.35)	110.78 (128.51)	14.31 (18.56)	33.77 (37.84)	80.61 (93.36)	18.20 (21.02)
		77.98 (91.81)			75.69 (94.27)			62.06 (76.18)	
Dialysis*	52.68 (61.80)	131.05 (160.98)	45.05 (57.37)	59.59 (69.82)	111.48 (130.13)	46.299 (59.89)	50.92 (56.89)	86.55 (98.12)	45.34 (53.68)
		131.05 (160.98)			111.48 (130.13)			86.55 (98.12)	

### Relation Between Travel Time and Social Indicators

To assess the disparity in weighted travel times among these countries, we compared the numbers of institutions that offer the related pediatric (inpatient, nephro-urology outpatient) services and correlated them with the respective country‘s social indicators population, land area, number of doctors working in the health care system, number of live births, and total health care expenditure (Supplementary Table 3). In Germany, there are more hospitals providing a pediatric inpatient service and more hemodialysis units than in Ireland or the UK, whereas the UK has the highest number of institutions with pediatric urology and pediatric nephrology outpatient services. The number of hospitals with a pediatric inpatient service correlated with social indicators does not yield an explanation for the differences in the weighted travel time to a pediatric inpatient ward between the countries. In contrast, a higher number of facilities with a pediatric nephrology outpatient service relative to population, land area, number of doctors, live births, and total health care expenditure is associated with shorter weighted travel times from the random points to the nearest pediatric nephrology outpatient service in the three countries. This, however, is less applicable to the number of institutions with a pediatric urology outpatient service and to the number of pediatric hemodialysis units in each country.

### Non-weighted Travel Time to Nearest Point of Service

**Figure 3:**
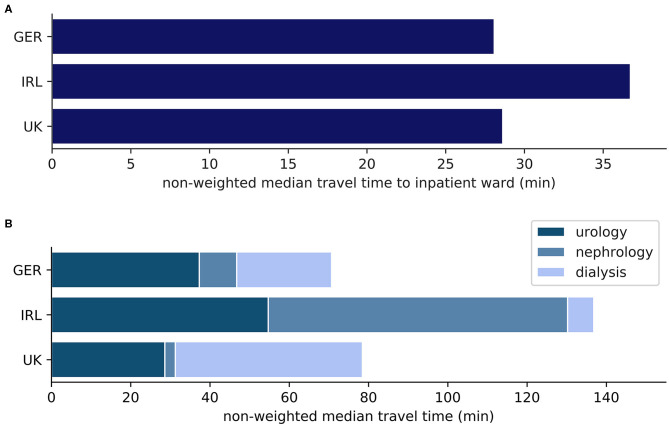
**(A)** Non-weighted mean travel time (min) from random points to nearest pediatric inpatient ward; **(B)** Non-weighted mean travel time (min) from random points to nearest facility with pediatric nephro-urology service; inpatient: pediatric inpatient ward; urology: pediatric urology outpatient clinic; nephrology: pediatric nephrology outpatient clinic; dialysis: pediatric hemodialysis unit.

**Figure 4:**
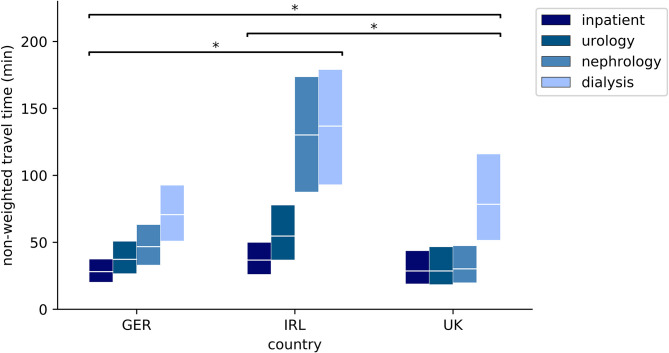
Non-weighted quartiles of travel time (min) from random points to nearest facility with pediatric inpatient or nephro-urology service (upper box edge: 75th percentile, middle line: median; lower box edge: 25th percentile); *p < 0.0001; inpatient: pediatric inpatient ward; urology: pediatric urology outpatient clinic; nephrology: pediatric nephrology outpatient clinic; dialysis: pediatric hemodialysis unit.

Weighting by population density favors urban regions with a high population density. If the aim of a health care system is to provide specialized services also in less inhabited regions rather than to focus on urban areas, such weighting may therefore be inappropriate. To address this, we utilized non-weighted results (Table 3, Figures 3, 4, Supplementary Table 4). In contrast to the weighted results discussed above, non-weighted travel times from the random points to the nearest pediatric inpatient ward and the nearest pediatric hemodialysis unit are shortest in Germany. As with the weighted results, non-weighted travel times to the nearest urology or nephrology outpatient clinic are shortest in the UK and non-weighted travel times to any of the nearest pediatric inpatient or nephro-urology service providers are longest in Ireland. Each of the observed differences between the countries is highly significant also for the non-weighted travel times (*p* < 0.0001 according to both Kruskal-Wallis test and Mann-Whitney *U*-test).

**Table 3:** Non-weighted median values, mean values, and standard deviation of travel time by car (min) from random points to nearest facility with pediatric service (inpatient: inpatient ward; urology: urology outpatient clinic; nephrology: nephrology outpatient clinic; dialysis: hemodialysis unit); Ireland (boxes): privately funded hospitals excluded (upper), included (lower); *p < 0.0001.

	**Median (min)**			**Mean (min)**			**Standard deviation (min)**		
**Country**	**GER**	**IRL**	**UK**	**GER**	**IRL**	**UK**	**GER**	**IRL**	**UK**
Inpatient*	28.08	36.73	28.62	29.72	39.77	36.77	13.19	19.67	28.94
		36.46			39.57			19.75	
Urology*	37.30	54.68	28.60	40.32	60.22	37.78	19.23	31.80	29.50
		51.04			56.80			29.78	
Nephrology*	46.73	130.21	31.24	30.21	131.60	38.77	22.97	55.59	28.92
		97.67			112.78			60.24	
Dialysis*	70.66	136.82	78.44	73.61	136.99	93.47	30.67	55.97	60.99
		136.82			136.99			55.97	

## Discussion

### The Results

The results of this study show a disparity between the geographical accessibility of institutions that provide specialized pediatric services in three industrialized European countries. Travel times to the nearest pediatric inpatient wards as well as to the nearest pediatric nephro-urology services weighted by population density are shortest in the UK, longer in Germany, and longest in Ireland.

This disparity is not in line with the inter-country differences in macroeconomic indicators (gross domestic product (GDP) per capita, total health expenditure per capita, or its amount of the GDP; data not shown). The results match better with other country-related characteristics. Firstly, Ireland's considerably lower population density (70.0/km^2^) compared to the UK (272.4/km^2^) or Germany (234.0/km^2^) probably is a main reason for longer travel times. Secondly, the countries exhibit different types of population distribution. The fraction of the population living in urban areas is highest in the UK (83%), lower in Germany (77%), and lowest in Ireland (63%), according to the World Bank. The population density of NUTS 3 regions is widely distributed in the UK, whereas more narrowly scattered in Germany (Supplementary Figure 3). This seems to be reflected by the contrast between shorter weighted, yet longer non-weighted travel times to the nearest inpatient ward and hemodialysis unit in the UK, compared to Germany. Lower non-weighted standard deviations of travel times to all types of nephro-urology service in Germany than in the UK may also be related to this. However, standard deviations of travel times being lower in Germany than in the UK may underline the German health care system's focus on a broader availability of health care services also in less inhabited regions. The distribution of population density among the NUTS 3 regions in Ireland is distorted mainly by its capital. Dublin, Ireland's largest congested urban area, naturally holds both public Irish hospitals that provide pediatric nephrology outpatient clinics and the only pediatric hemodialysis unit in Ireland. Yet it is located far from the geographical center of Ireland, which seems to be a reason for longer travel times in Ireland than in the two other countries.

Apart from widely fixed country-related factors such as the population density and the distribution of the population, there may be alterable reasons for differing travel times from assumptive patients' homes to providers of pediatric nephro-urology services among the countries. Clearly an important reason for both the weighted and non-weighted travel times to the nearest pediatric urology and nephrology outpatient clinics in the UK being the lowest among the three countries is the high number per capita of institutions that provide these services. The ratio of the number of facilities with pediatric nephro-urology services to any of the country-related figures is highest in the UK by far, although the number of doctors working in the health care system (relative to the population) is lowest in the UK (Supplementary Table 3). This seems to be related to two facts: Firstly, in the UK hospitals are integrated in regional NHS Trusts together with other institutions. This facilitates the availability of pediatric nephro-urology services in more than one institution of the same NHS Trust. Secondly, in the UK there are networks of pediatric nephrology centers and local pediatric departments. These networks involve both outreach clinics in local facilities that are held by staff of the pediatric nephrology centers, and further training of local pediatricians, who then offer their own pediatric nephrology services. We do not know of clinical networks in pediatric nephrology of this extent in Germany or Ireland (the Gesellschaft für Pädiatrische Nephrologie is an active network of German-speaking pediatric nephrologists, however does not provide structures for local outreach clinics or education for local pediatricians). This is probably another main explanation for travel times from assumptive patients' homes to pediatric nephrology outpatient clinics in the UK being shorter than in Germany or Ireland. This observation may encourage health administrators to establish networks of pediatricians, as they increase the geographical accessibility of specialized pediatric health services without the necessity to increase the number of working pediatricians. In addition to outreach clinics, such networks may involve telemedicine, which was successfully introduced in the field of pediatric nephrology in Queensland, Australia (17). While there is a natural demand for telehealth services in Queensland due the low population density, telemedicine may reduce patients' travel burden also in countries with a higher population density such as Germany, Ireland, or the UK.

Considering our clinical experience that patients living far from a tertiary hospital are more likely to miss an appointment or discontinue the treatment than those living closer, the clinical relevance of the observed difference in travel times to specialized pediatric nephro-urology care between the three countries seems obvious. However, as mentioned above, the data published to support this thesis is very limited and more research into this field is required.

### The Model

This study introduces a model to compare countries or regions regarding travel distance and time from patients' homes to health service providers. The model uses random geographical points within one country (a virtual representation of patients' homes) as starting points, and geographical coordinates of the real service providers (hospitals, clinics) as destinations. To address the uneven distribution of the population within the countries, the results are weighted by the population density of the NUTS 3 region that the assumptive patients' homes belong to.

As this model is based on publicly available data (location of health service providers and regional population density) and utilizes only publicly accessible applications, it can be easily and rapidly applied to any health service in any country by anyone who is capable of programming with Python, the most popular programming language nowadays according to PYPL index. In addition to comparing travel time and distance between countries or regions, the Python script we have established can be easily adapted to highlight gaps in the distribution of health services within a country or region. This can help health administrators to adjust networks of health services. The Python script we have used in this study is available from the corresponding author on request.

To keep calculations feasible and easily transferable, the model is based merely on geographical location of health institutions and regional population density. Thus, it does not include the supply and demand aspect of service provision (e.g., catchment population or temporal availability of services). In this study, we used NUTS 3 regions as the smallest available unit that (by its population density) determines the weighting of the results. However, the population density within such regions may be non-homogenous, possibly leading to results slightly differing from reality. As the relation of geodesic distances from the virtual patients' homes to the nearest service providers among the three countries is in line with the corresponding travel times, the disparities between the travel times among Germany, Ireland, and the UK we have shown here are not explained directly by the road infrastructure. Traffic congestion, however, may influence travel times. According to INRIX and TomTom traffic indices, Dublin is the most congested among the cities that hold a pediatric hemodialysis unit (Supplementary Table 5). Of the ten cities with a pediatric hemodialysis unit and the highest traffic congestion indices, six ones belong to the UK and only three of the cities belong to Germany. This indicates that the actual difference between travel times to a hemodialysis unit in Ireland and the two other countries might be even more pronounced, whereas the actual difference between weighted travel times to a hemodialysis unit in Germany and the UK might be less distinct. However, as severe traffic congestion may be expected only in major congested urban areas, it presumably only has a minor effect on the actual travel times to services with a broader availability than pediatric hemodialysis, such as general inpatient services (in Germany, Ireland, and the UK) and specialized urology/nephrology outpatient services (in Germany and the UK).

## Conclusions

Our data shows a disparity between the travel times from assumptive patients' homes to the nearest pediatric inpatient ward and the nearest points of pediatric nephro-urology service in the three industrialized European countries Germany, Ireland, and the UK. Reasons for this may be differences in the overall population density, in the distribution of the population across the country, and in the focus of the health care systems. Furthermore, clinical networking within the field of pediatric nephrology in the UK seems to account for travel times in the UK being the shortest among the three countries. The model that is introduced in this study involves only publicly accessible data and applications and may easily be employed to compare travel times and distances in further countries or regions.

## Data Availability Statement

The raw data supporting the conclusions of this article will be made available by the authors, without undue reservation.

## Author Contributions

NT conceptualized and designed the study, collected data, designed instruments for the data analyses, carried out the data analyses, drafted the initial manuscript, and reviewed and revised the manuscript. DL provided the initial instruments for the data analyses, supervised the design of further instruments for the data analyses, supervised the data analyses, and reviewed and revised the manuscript. AK, MK, and MT supervised the data analyses, and reviewed and revised the manuscript. MM helped to prepare the analyses for publication, and critically reviewed and revised the manuscript. KD conceptualized the study, supervised the data analyses, and critically reviewed and revised the manuscript. All authors contributed to the article and approved the submitted version.

## Conflict of Interest

KD was employed by the non-profit organization Deutsche Stiftung Organtransplantation. The remaining authors declare that the research was conducted in the absence of any commercial or financial relationships that could be construed as a potential conflict of interest.
