# Supplementary material for: Geographical Accessibility of Pediatric Inpatient, Nephrology, and Urology Services in Europe
Source: Front Pediatr. 2020 Jul 21;8:395. doi: 10.3389/fped.2020.00395 (PMC7396594; doi:10.3389/fped.2020.00395)
Supplement: Supplementary file 1 [file Data_Sheet_1.PDF]

Supplementary Figures

Supplementary Figure 1

a

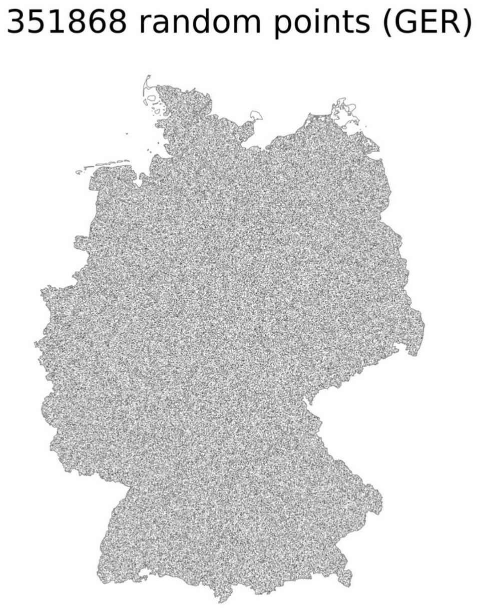

b

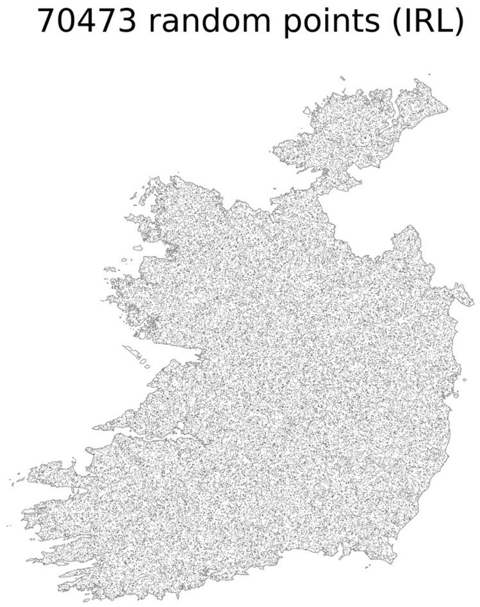

c

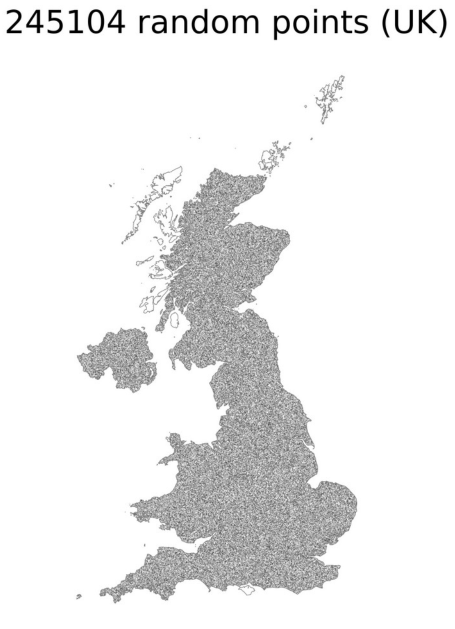

Suppl. Fig. 1 a, Distribution of 351,868 random points (grey) in Germany; b, Distribution of 70,473 random points (grey) in Ireland; c, Distribution of 245,104 random points (grey) in the United Kingdom. Coordinate reference system: EPSG:3035

Supplementary Figure 2

a

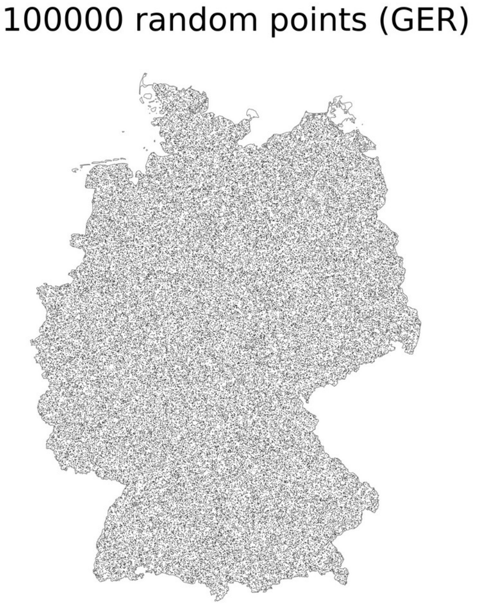

b

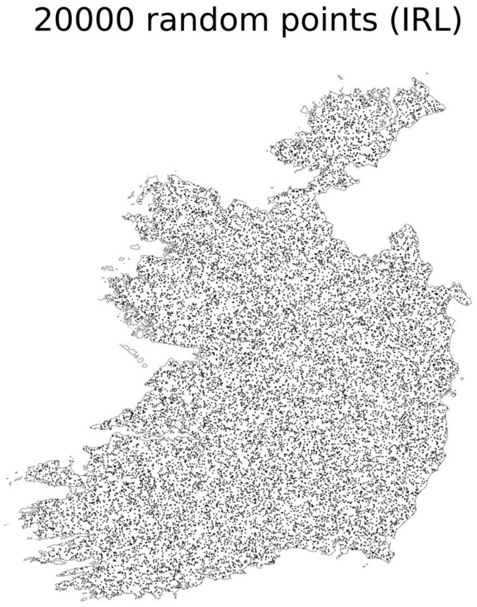

c

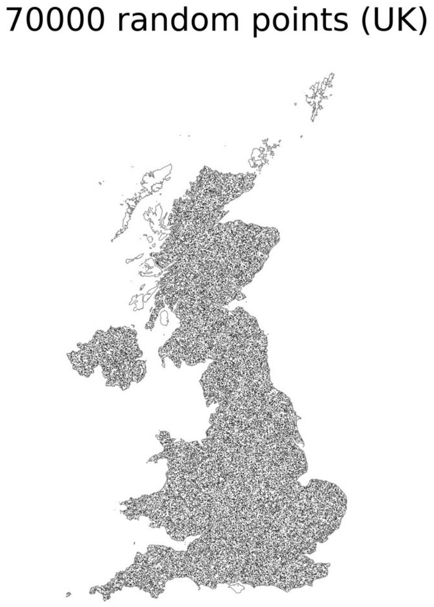

Suppl. Fig. 2 a, Distribution of 100,000 random points (grey) in Germany; b, Distribution of 20,000 random points (grey) in Ireland; c, Distribution of 70,000 random points (grey) in the United Kingdom. Coordinate reference system: EPSG:3035

Supplementary Figure 3

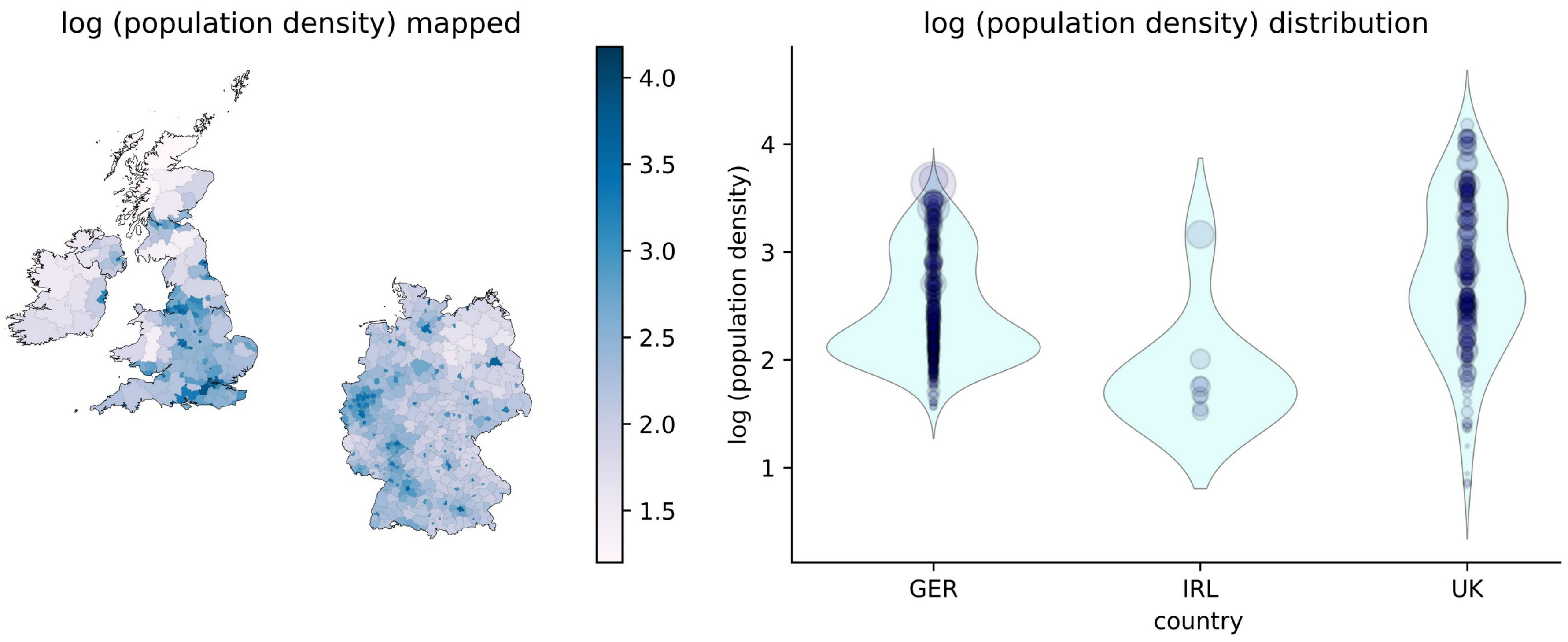

Suppl. Fig. 3 Left, Logarithm (base 10) of population density of NUTS 3 regions, indicated by color intensity, mapped; Coordinate reference system: EPSG:3035; Right, Distribution of population density of NUTS 3 regions; scatter plot (x axis: country; y axis: logarithm (base 10) of population density) with each NUTS 3 region represented by one marker and marker size indicating population of NUTS 3 region; markers are semi-transparent, with color intensity indicating overlay of markers; violin plot with width indicating distribution of markers along y axis

Supplementary Tables

Supplementary Table 1

	GER	IRL	UK
inpatient	hospital with general pediatric inpatient ward open 24h per day,7days per week and lead by department of pediatrics	hospital with general pediatric inpatient ward open 24h per day,7days per week and lead by department of pediatrics	hospital with general pediatric inpatient ward open 24h per day,7days per week and lead by department of pediatrics
urology	designated pediatric urology outpatient clinic (lead by department of general surgery, pediatric surgery, or urology)	designated pediatric urology outpatient clinic (lead by department of general surgery, pediatric surgery, or urology)	designated pediatric urology outpatient clinic (lead by department of general surgery, pediatric surgery, or urology)
	outpatient clinic lead by pediatric surgeon with designated experience in urology	outpatient clinic lead by pediatric surgeon with designated experience in urology	outpatient clinic lead by pediatric surgeon with designated experience in urology
		hospital with >5 patients on National Treatment Purchase Fund „OP Waiting List By Group Hospital 2018“ in any month („Specialty“: „Urology“; „Age Profile“: „0-15“; „Time Band“: „0-3 Months“)	hospital with designated pediatric urology outpatient clinic in eReferral service (according to February 2019 to April 2019 EBSX05 reports from digital.nhs.uk/)
	designated pediatric nephrology outpatient clinic	designated pediatric nephrology outpatient clinic	designated pediatric nephrology outpatient clinic
	outpatient clinic lead by pediatric nephrology consultant	outpatient clinic lead by pediatric nephrology consultant	outpatient clinic lead by pediatric nephrology consultant
	outpatient clinic lead by pediatrician with special interest in pediatric nephrology	outpatient clinic lead by pediatrician with special interest in pediatric nephrology	outpatient clinic lead by pediatrician with special interest in pediatric nephrology

		hospital with >5 patients on national treatment purchase fund „OP Waiting List By Group Hospital 2018“ in any month („Specialty“: „Paed Nephrology“ or „Nephrology“; „Age Profile“: „0-15“; „Time Band“: „0-3 Months“)	shared care outpatient clinic with visiting pediatric nephrology consultant
			hospital with designated paediatric nephrology outpatient clinic in eReferral service (according to February 2019 to April 2019 EBSX05 reports from digital.nhs.uk/)
nephrology	designated pediatric nephrology outpatient clinic	designated pediatric nephrology outpatient clinic	designated pediatric nephrology outpatient clinic
	outpatient clinic lead by pediatric nephrology consultant	outpatient clinic lead by pediatric nephrology consultant	outpatient clinic lead by pediatric nephrology consultant
	outpatient clinic lead by pediatrician with special interest in pediatric nephrology	outpatient clinic lead by pediatrician with special interest in pediatric nephrology	outpatient clinic lead by pediatrician with special interest in pediatric nephrology
		hospital with >5 patients on national treatment purchase fund „OP Waiting List By Group Hospital 2018“ in any month („Specialty“: „Paed Nephrology“ or „Nephrology“; „Age Profile“: „0-15“; „Time Band“: „0-3 Months“)	shared care outpatient clinic with visiting pediatric nephrology consultant
			hospital with designated paediatric nephrology outpatient clinic in eReferral service (according to February 2019 to April 2019 EBSX05 reports from digital.nhs.uk/)
dialysis	pediatric hemodialysis unit	pediatric hemodialysis unit	pediatric hemodialysis unit

Suppl. Table 1 Inclusion criteria for facilities that provide a type of pediatric service (inpatient: inpatient ward; urology: urology outpatient clinic; nephrology: nephrology outpatient clinic; dialysis: hemodialysis unit); to be classified as providing the type of service, a facility had to fulfill ≥1 inclusion criterion

Supplementary Table 2

	GER	IRL	UK
inpatient	hospital not providing acute services	hospital not providing acute services	hospital not providing acute services
	neonatology department without pediatric services for non-neonates	neonatology department without pediatric services for non-neonates	neonatology department without pediatric services for non-neonates
	hospital with pedatric psychiatry department without department of (somatic) pediatrics	hospital with pedatric psychiatry department without department of (somatic) pediatrics	hospital with pedatric psychiatry department without department of (somatic) pediatrics
	pediatric department covering only one pediatric specialty (not nephrology)	pediatric department covering only one pediatric specialty (not nephrology)	pediatric department covering only one pediatric specialty (not nephrology)
urology	private practice without link to inpatient department	private practice without link to inpatient department	private practice without link to inpatient department
	outpatient clinic (at least partly) not covering age range: 0 to 12 years	outpatient clinic (at least partly) not covering age range: 0 to 12 years	outpatient clinic (at least partly) not covering age range: 0 to 12 years
	outpatient clinic covering only enuresis and/or incontinence	outpatient clinic covering only enuresis and/or incontinence	outpatient clinic covering only enuresis and/or incontinence
			exclusion of „complex“ cases in eReferral service (according to February 2019 to April 2019 EBSX05 reports from digital.nhs.uk/)
			exclusion of female or male gender in eReferral service (according to February 2019 to April 2019 EBSX05 reports from digital.nhs.uk/)
nephrology	private practice without link to inpatient department	private practice without link to inpatient department	private practice without link to inpatient department
	outpatient clinic (partly) not covering age range: 0 to 12 years	outpatient clinic (partly) not covering age range: 0 to 12 years	outpatient clinic (partly) not covering age range: 0 to 12 years
	outpatient clinic covering only enuresis and/or incontinence	outpatient clinic covering only enuresis and/or incontinence	outpatient clinic covering only enuresis and/or incontinence

dialysis	(none)	(none)	(none)

Suppl. Table 2 Exclusion criteria for facilities that do not provide a type of pediatric service (inpatient: inpatient ward; urology: urology outpatient clinic; nephrology: nephrology outpatient clinic; dialysis: hemodialysis unit); to be classified as not providing the type of service, a facility had to fulfill ≥1 exclusion criterion

Supplementary Table 3

	inpatient			urology			nephrology			dialysis		
country	GER	IRL	UK	GER	IRL	UK	GER	IRL	UK	GER	IRL	UK
number	341	22	182	181	11	207	83	2	169	19	1	13
		25			15			3			1	
per 10^6 population[a]	4.12	4.55	2.75	2.19	2.28	3.12	1.00	0.41	2.55	0.23	0.21	0.20
		5.18			3.11			0.62				
per 10^3 km² area[b]	0.95	0.32	0.73	0.51	0.16	0.83	0.23	0.03	0.68	0.05	0.01	0.05
		0.36			0.21			0.04				
per 10^3 doctors[c]	0.99	1.45	0.99	0.53	0.72	1.13	0.24	0.13	0.92	0.06	0.07	0.07
		1.65			0.99			0.20				
per 10^3 live births[d]	0.43	0.36	0.24	0.23	0.18	0.27	0.11	0.03	0.22	0.02	0.02	0.02
		0.40			0.24			0.05				
per 10^6 € total health care expenditure[e]	0.97	1.08	0.78	0.52	0.54	0.89	0.24	0.10	0.72	0.05	0.05	0.06
		1.23		0.74				0.15				

Suppl. Table 3 Number of facilities that provide a type of pediatric service (inpatient: inpatient ward; urology: urology outpatient clinic; nephrology: nephrology outpatient clinic; dialysis: hemodialysis unit), absolute and per country-related figures; Eurostat data from [a]2018; [b]2015; [c]2016; [d]2017; [e]2016; Ireland (boxes): privately funded hospitals excluded (upper), included (lower)

Supplementary Table 4

	median (km)			mean (km)			standard deviation (km)		
country	GER	IRL	UK	GER	IRL	UK	GER	IRL	UK
inpatient*	19.73	35.38	26.27	21.01	35.68	35.95	11.13	16.33	31.21
		35.26			35.54			16.32	
urology*	29.47	54.54	26.28	31.87	60.64	39.86	17.72	34.54	38.06
		50.13			53.42			27.22	
nephrology*	41.44	234.79	28.11	44.32	227.42	40.89	24.06	109.14	37.60
		128.35			137.75			71.20	
dialysis*	78.70	239.83	90.51	83.41	233.05	106.09	42.80	109.89	67.01
		239.83			233.05			109.89	

Suppl. Table 4 Non-weighted median values, mean values and standard deviation of geodesic distance (km) from random points to nearest facility with pediatric service (inpatient: inpatient ward; urology: urology outpatient clinic; nephrology: nephrology outpatient clinic; dialysis: hemodialysis unit); Ireland (boxes): privately funded hospitals excluded (upper), included (lower); *$p < 0.0001$

Supplementary Table 5

city	country	INRIX hours 2019[a]	INRIX rank	TomTom %[b] 2019	TomTom rank	combined rank
Dublin	Ireland	154	1	48	1	1
London	UK	149	2	38	2	2
Belfast	UK	112	3	33	4	3
Bristol	UK	103	4	33	4	4
Manchester	UK	92	5	33	4	5
Southampton	UK	79	9	33	4	6
Cardiff	UK	87	6	30	9	7
Munich	Germany	87	6	30	9	7
Hamburg	Germany	48	15	34	3	9
Berlin	Germany	66	11	32	8	10
Birmingham	UK	80	8	28	13	11
Nottingham	UK	78	10	27	15	12
Stuttgart	Germany	42	17	30	9	13
Leeds	UK	66	11	27	15	14
Liverpool	UK	52	14	28	13	15
Newcastle	UK	57	13	25	19	16
Bonn	Germany	35	22	29	12	17
Glasgow	UK	43	16	25	19	18
Frankfurt	Germany	36	21	27	15	19
Cologne	Germany	41	18	26	18	20
Freiburg	Germany	38	20	23	22	21
Hannover	Germany	40	19	21	24	22
Leipzig	Germany	33	24	24	21	23

Essen	Germany	29	25	23	22	24
Muenster	Germany	34	23	21	24	25
Marburg	Germany	16	26	N	26	26
Heidelberg	Germany	N	27	N	26	27
Jena	Germany	N	27	N	26	27
Memmingen	Germany	N	27	N	26	27
Rostock	Germany	N	27	N	26	27
Tuebingen	Germany	N	27	N	26	27
Erlangen	Germany	N	27	N	26	27

Suppl. Table 5 Traffic congestion indices of cities with pediatric hemodialysis unit; cities sorted by combined rank in ascending order; [a]INRIX hours lost in congestion 2019; [b]TomTom congestion level 2019
